# Psychological well-being and its associations with sociodemographic characteristics, physical health, substance use and other mental health outcomes among adults in Canada

**DOI:** 10.24095/hpcdp.44.10.03

**Published:** 2024-10

**Authors:** Melanie Varin, Zahra M. Clayborne, Melissa M. Baker, Elia Palladino, Heather Orpana, Colin A. Capaldi

**Affiliations:** 1 Centre for Surveillance and Applied Research, Public Health Agency of Canada, Ottawa, Ontario, Canada; 2 School of Epidemiology and Public Health, University of Ottawa, Ontario, Canada; 3 Helen Keller International, Nairobi, Kenya; 4 Centre for Immunization and Respiratory Infectious Diseases, Public Health Agency of Canada, Ottawa, Ontario, Canada

**Keywords:** psychological well-being, positive mental health, mental illness, sociodemographic characteristics, substance use, physical health, population health

## Abstract

**Introduction:**

Psychological well-being (PWB) is an important component of positive mental health (PMH) and an asset for population health. This study examined correlates of PWB among community-dwelling adults (18+ years) in the 10 Canadian provinces.

**Methods:**

Using data from the 2019 Canadian Community Health Survey Rapid Response on PMH, we conducted linear regression analyses with sociodemographic, mental health, physical health and substance use variables as predictors of PWB. PWB was measured using six questions from the Mental Health Continuum—Short Form, which asked about feelings of self-acceptance, personal growth, environmental mastery, autonomy, positive relations and purpose in life during the past month.

**Results:**

In unadjusted and adjusted analyses, older age, being married or in a common-law relationship and having a BMI in the overweight category (25.00–29.99) were associated with higher PWB, while reporting a mood disorder, anxiety disorder, high perceived life stress, engaging in heavy episodic drinking and frequent cannabis use were associated with lower PWB. Sex, having children living at home, immigrant status, racialized group membership, educational attainment, household income tertile, having a BMI in the obese category (≥30.00), major chronic disease and smoking status were not significantly associated with PWB.

**Conclusion:**

This research identifies sociodemographic, mental health, physical health and substance use factors associated with PWB among adults in Canada. These findings highlight groups and characteristics that could be the focus of future research to promote PMH.

**Highlights**

- This study examined psychological well-being and its associations with sociodemographic, mental health, physical health and substance use factors among individuals aged 18 years and older in Canada.
- Older age, being married or in common-law relationship, and having a BMI in the overweight category were associated with higher psychological well-being.
- Reporting a mood disorder, an anxiety disorder, high perceived life stress, engaging in heavy episodic drinking and frequent cannabis use were associated with lower psychological well-being.

## Introduction

The promotion of well-being is a public health priority in both Canada and globally, as evidenced in national and international mental health strategies.^1^,^2^ While well-being and related concepts have increasingly received attention by researchers internationally and over time,^3^ some have argued that the primary focus of epidemiological and public health research on disease states and their risk factors need to be supplemented with research on positive psychological states and health assets to gain a complete understanding of population health.^4^

Hedonia and eudaimonia represent two distinct components of well-being.^5^ While both are components of positive mental health (PMH) and are related, they can have unique psychological, social and biological correlates.^6^-^8^ Hedonia typically covers aspects of feeling good, including experiencing positive emotions and satisfaction with life.^5^ In contrast, eudaimonia can be defined as functioning well,^5^ which is captured in Ryff’s concept of psychological well-being (PWB) as including self-acceptance, personal growth, purpose in life, environmental mastery, autonomy and positive relations with others.^9^

PWB has been associated with a range of health outcomes, for example, better subjective health, fewer sleep problems, lower levels of inflammation and reduced cardiovascular events such as myocardial infarctions.^8^ Some aspects of PWB (e.g. purpose in life) have also been associated with reduced risk of all-cause mortality.^8^,^10^ Within Canada, previous research has found that individuals who report higher PWB tend to report better well-being on other measures of PMH (e.g. life satisfaction), as well as less distress and fewer functional limitations.^11^ Moreover, negative bivariate associations have been found between PWB and alcohol, cannabis and cigarette use among youth in Canada.^12^,^13^

In 2016, the Public Health Agency of Canada (PHAC) released the Positive Mental Health Surveillance Indicator Framework as a tool to monitor and report on PMH outcomes and associated risk and protective factors in the Canadian population;^14^,^15^ PWB was identified as a key PMH outcome in the Framework. Given the importance of mental health promotion nationally and internationally, identifying factors associated with PWB is essential to build upon previous findings, inform continued surveillance and identify groups who may benefit from interventions that promote higher PWB. Thus, the objective of this study was to examine associations between PWB and a range of sociodemographic, mental health, physical health and substance use variables among adults in Canada.

## Methods

***Study design and population***

This secondary data analysis used the 2019 Canadian Community Health Survey (CCHS) Rapid Response on PMH. The CCHS is a voluntary cross-sectional survey, conducted annually by Statistics Canada, of individuals aged 12 years and older living in all the Canadian provinces and territories.^16^ The survey coverage excluded individuals who were institutionalized or living in foster homes, full-time members of the Canadian Forces, and individuals living on reserves and in other Indigenous settlements in the provinces and in two specific health regions in Quebec; these exclusions represent less than 3% of the Canadian population.^16^ Data for the Rapid Response on PMH were collected between January and March 2019 from non-proxy respondents living in the 10 Canadian provinces, with a response rate of 58.3%. We limited our analyses to adults aged 18 years and older (N = 11486).

***Measures***

**Psychological well-being**

PWB was measured using six questions from the Mental Health Continuum—Short Form.^17^ These asked about feelings of self-acceptance, personal growth, environmental mastery, autonomy, positive relations with others and purpose in life in the past month. In accordance with the adult Positive Mental Health Surveillance Indicator Framework^15^ and previous research,^11^,^18^ response options for each question were converted into number of days in the past month as follows: “every day” as 28 days (4 weeks 7 days per week); “almost every day” as 20 days (4 weeks 5 days per week); “about 2 or 3 times a week” as 10 days (4 weeks 2.5 days per week); “about once a week” as 4 days (4 weeks 1 day per week); “once or twice” as 1.5 days; and “never” as 0 days. Mean scores were generated by summing item scores and dividing by the total number of items; scores ranged from 0 to 28. The Mental Health Continuum—Short Form PWB subscale has been validated in previous Canadian population health surveys^11^ and had an acceptable internal consistency in the Rapid Response dataset (Cronbach α= 0.79).

**Sociodemographic variables**

We examined a number of sociodemographic characteristics: sex (male, female); age (continuous); marital status (married/in a common-law relationship, single/separated/divorced/widowed); household income tertile (low, middle, high); respondent’s highest level of education (high school graduation or less, postsecondary graduation); immigrant status (yes [landed immigrant/non–permanent resident], no [Canadian-born]); racialized group member (yes [identified as Indigenous or any racialized background], no [only identified as White]); and household composition (children of any age living at home, no children living at home). Income data were based on a combination of tax data (~20%), collected respondent data (~15%) and imputed income amounts (~65%).

**Mental health variables**

Perceived life stress was examined using the question “Thinking about the amount of stress in your life, would you say that most of your days are…?” Response options included “not at all stressful,” “not very stressful,” “a bit stressful,” “quite a bit stressful” and “extremely stressful.” Consistent with the PHAC Suicide Surveillance Indicator Framework,^19^ respondents who answered “extremely stressful” or “quite a bit stressful” were categorized as having high perceived life stress.

Presence of a mood disorder was assessed with the question “Do you have a mood disorder such as depression, bipolar disorder, mania or dysthymia?” Presence of an anxiety disorder was assessed with the question “Do you have an anxiety disorder such as a phobia, obsessive-compulsive disorder or a panic disorder?” Respondents who answered “yes” to the first question were categorized as having a mood disorder and “yes” to the second question as having an anxiety disorder.

**Physical health variables**

The derived body mass index (BMI) variable (HWTDVBCC) created by Statistics Canada was modified to report on BMI. This derived variable was calculated based on the self-reported height and weight of the respondent (excluding those who reported being pregnant or did not answer the pregnancy question when asked). Sex-specific corrections were applied to adjust for the tendency of individuals to overestimate their height and underestimate their weight.^20^ After these adjustments, respondents’ BMI were classified as normal/underweight (≤24.99), overweight (25.00–29.99) or obese (≥30.00) for analysis.^21^ The normal weight and underweight category groups were combined because the percentage of individuals with a BMI classified as underweight was low, and the interpretation of results was similar when the two groups were examined separately in sensitivity analyses.

Consistent with the Suicide Surveillance Indicator Framework,^19^ respondents were categorized as having at least one major chronic disease if they reported having asthma; chronic bronchitis, emphysema or chronic obstructive pulmonary disease; heart disease; diabetes; a current or previous cancer diagnosis; or if they experienced the effects of a stroke.

**Substance use variables**

Current smoking status was assessed with the question “At the present time, do you smoke cigarettes every day, occasionally or not at all?”

Heavy episodic drinking was based on the 2018 *Canada’s low-risk alcohol drinking guidelines*^22^ and was assessed using the question “How often in the past 12 months have you had 5 (male) / 4 (female) or more drinks on one occasion?” Response options ranged from “never” to “more than once a week.” Individuals who indicated “never” or who reported not having an alcoholic drink during the past 12 months were categorized as not engaging in heavy episodic drinking in the past year, while those who reported drinking that many drinks “less than once a month” or more often were categorized as engaging in heavy episodic drinking in the past year.

Cannabis use was assessed using the question “How often did you use cannabis in the past 12 months?” Based on *Canada’s Lower-risk Cannabis Use Guidelines* relating to frequency and intensity of use,^23^ respondents who indicated that they used cannabis “more than once a week” or “daily or almost daily” were categorized as engaging in frequent cannabis use, while those who responded “once a week” or less frequently were categorized as not engaging in frequent cannabis use.

***Statistical analyses***

We conducted analyses using SAS Enterprise Guide version 7.1 (SAS Institute, Cary, NC, US). Descriptive statistics were reported using weighted percentages, means and medians with 95% confidence intervals (CIs). To account for the complex survey design, we calculated 95% CIs using the bootstrap resampling method with 1000 replications. Survey sampling weights and bootstrap weights were provided by Statistics Canada. We conducted unadjusted linear regression analyses to examine the bivariate association between each potential explanatory variable and PWB. We then conducted an adjusted linear regression analysis with variables that were statistically significant in the unadjusted analyses to simultaneously examine how each variable was related to PWB when adjusting for the other variables. To ensure that the sample composition remained consistent for unadjusted and adjusted results, all regression analyses were restricted to the 87.0% of respondents (N = 9993) with complete data on all relevant variables. Regression coefficients with *p* values less than 0.05 were interpreted as being statistically significant.

Multicollinearity did not appear to be an issue as none of the correlation coefficients between explanatory variables exceeded 0.60 and variance inflation factors in the adjusted model were all less than or equal to 1.43. These multicollinearity checks were weighted but bootstrapping was not applied for variance estimation due to SAS limitations. A residual plot was generated to confirm the assumption of homoscedasticity for the adjusted model.

## Results

***Descriptive statistics***

The majority of the population was married or in a common-law relationship (63.2%), had completed postsecondary education (64.1%), were non-immigrants (71.9%) and were not members of racialized groups (72.4%). Half of the individuals had children living at home (50.9%) (Table 1). 

**Table 1:** Descriptive characteristics of 2019 CCHS Rapid Response on PMH respondents (N = 11 486)

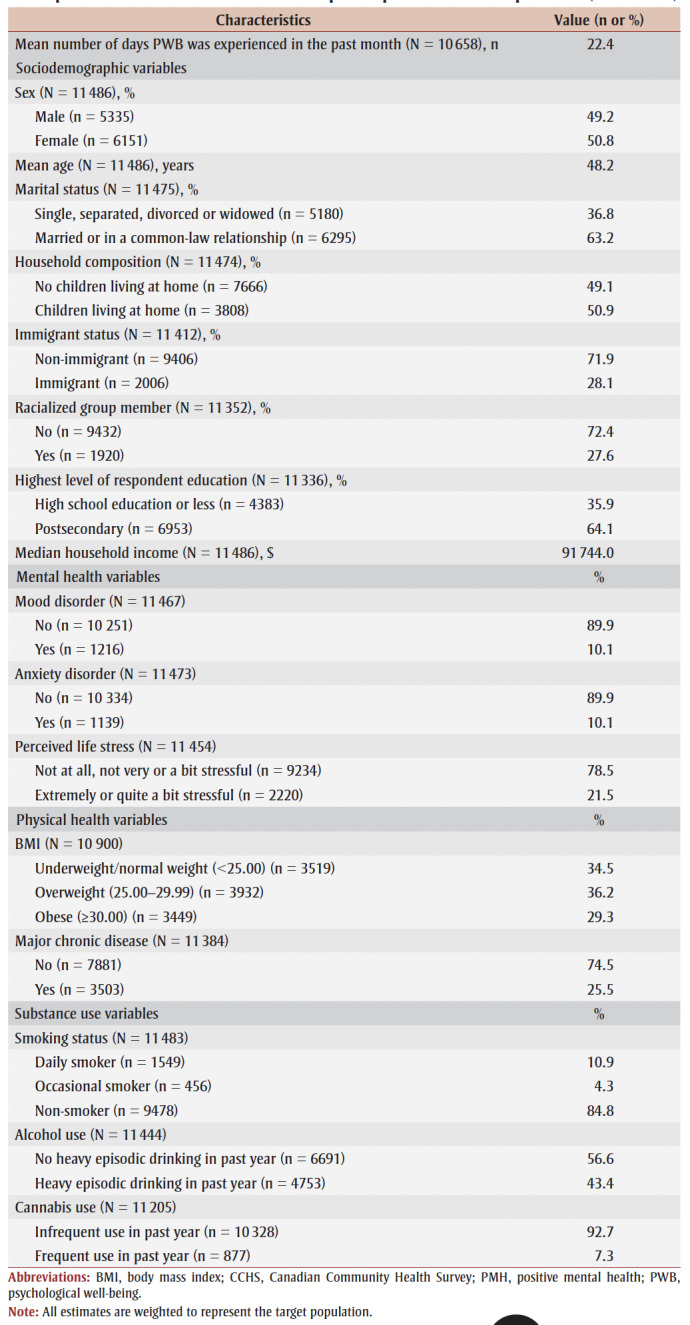

Most respondents did not report a mood (89.9%) or an anxiety (89.9%) disorder and had low perceived life stress (78.5%). The majority did not report having a major chronic disease (74.5%); however, around two-thirds were classified as having BMI in the overweight or obese category (65.5%). Most did not engage in frequent cannabis use (92.7%), were non-smokers (84.8%) and did not engage in heavy episodic drinking (56.6%) in the past year. 

Median household income before taxes was $91 744 and the mean age was 48.2 years. On average, PWB was experienced on 22.4 days in the past month.

***Linear regression results***

In the unadjusted analyses, older age (*B*= 0.03; 95% CI: 0.02 to 0.04), being married or in a common-law relationship (*B* = 1.12; 95% CI: 0.76 to 1.48) and having BMI in the overweight category (*B*=0.69; 95% CI: 0.28 to 1.10) were significantly associated with higher PWB. In contrast, reporting a mood disorder (*B* = −4.54; 95% CI: −5.25 to −3.83), an anxiety disorder (*B* = −3.45; 95% CI: −4.19 to −2.71), high perceived life stress (*B*=−1.53; 95% CI: −2.01 to −1.04), heavy episodic drinking in the past year (*B* = −0.69; 95%CI: −1.03, −0.34) and frequent cannabis use in the past year (*B* = −1.93; 95% CI: −2.61 to−1.24) were significantly associated with lower PWB in the unadjusted analyses. Sex, having children living at home, immigrant status, racialized group membership, educational attainment, household income tertile, having a BMI in the obese category (vs. underweight/normal weight category), having a major chronic disease and smoking status were not significantly associated with PWB in the unadjusted analyses (Table 2).

**Table 2:** Linear regression models examining associations between sociodemographic, mental health, physical health and substance use variables and PWB, 2019 CCHS Rapid Response on PMH (N = 9993)

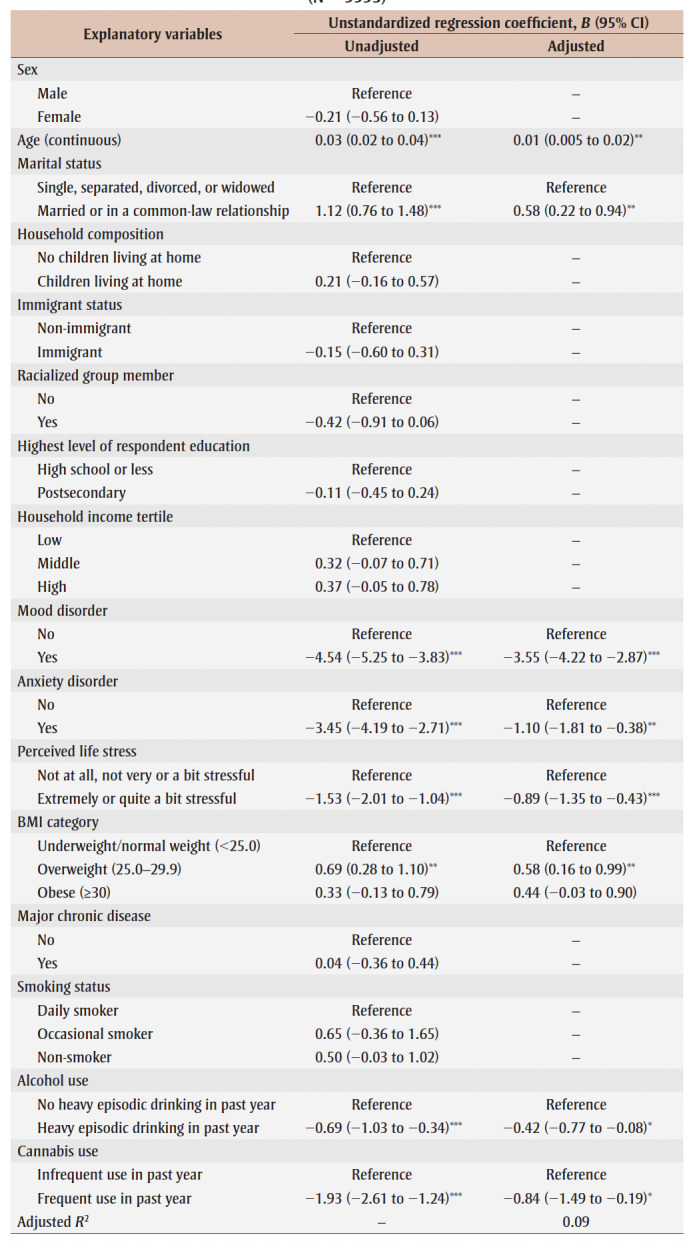 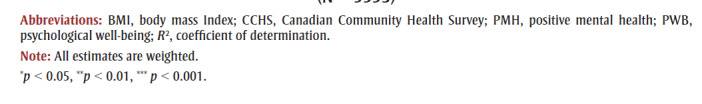

In the adjusted analyses, older age (*B*=0.01; 95% CI: 0.005 to 0.02), being married/in a common-law relationship (*B* = 0.58; 95% CI: 0.22 to 0.94) and having a BMI in the overweight category (*B* = 0.58; 95% CI: 0.16 to 0.99) were significantly associated with higher PWB, while reporting a mood disorder (*B* = −3.55; 95% CI: −4.22 to −2.87), anxiety disorder (*B* = −1.10; 95% CI: −1.81 to −0.38) or high perceived life stress (*B* = −0.89; 95% CI: −1.35 to −0.43) or engaging in heavy episodic drinking (*B* = −0.42; 95% CI: −0.77 to −0.08) and frequent cannabis use (*B*=−0.84; 95% CI: −1.49 to −0.19) were significantly associated with lower PWB.

## Discussion

In this study, we examined associations between PWB and sociodemographic characteristics, mental health, physical health and substance use using data from almost 10000 adults in Canada. We found that PWB was positively associated with two sociodemographic factors (i.e. age and being married or in a common-law relationship) and one physical health characteristic (i.e. having a BMI in the overweight category) and negatively associated with several mental health (i.e. reporting a mood disorder, an anxiety disorder and high perceived life stress) and substance use variables (i.e. engaging in heavy episodic drinking and frequent cannabis use in the past year). These associations with PWB were robust as they all remained statistically significant when simultaneously controlling for each other in the adjusted analysis.

Higher age was associated with higher PWB in the current study, which is consistent with previous Canadian findings showing that PMH outcomes are often most commonly observed among older adults.^15^,^18^,^24^ While we examined overall PWB, more detailed investigations into specific dimensions of PWB have found more nuanced patterns of results, with some aspects of PWB increasing as people age (e.g. environmental mastery) and other aspects decreasing (e.g. purpose in life).^25^ Future Canadian research could expand upon the current study by exploring different aspects of PWB and/or investigating correlates of PWB within developmental stages (early adulthood, mid-adulthood, older adulthood) to see if they might differ across the life course.

Marital status was also robustly associated with PWB. Longitudinal research has found overall increases in all six aspects of PWB when people are married for the first time, while some aspects of PWB decrease when people are separated/divorced (i.e. self-acceptance and positive relations) or widowed (i.e. purpose in life).^26^ Other longitudinal research indicates that the transition to cohabitation or marriage is generally associated with increases in happiness.^27^ There are many pathways through which long-term romantic relationships may promote PMH—for example, romantic partners can provide friendship, social support and emotional regulation.^28^,^29^

Having a BMI in the overweight category but not the obese category was consistently associated with a higher PWB score, which was unexpected but not idiosyncratic to this study. For instance, a meta-analysis found that mental health–related quality of life was higher among individuals with BMI in the overweight category (vs. normal category), although their physical health–related quality of life was lower.^30^ The literature on BMI and mental health more broadly can be inconsistent and nuanced, with associations sometimes depending on sex, age, stigma and measure of mental health.^31^,^32^ Studies are needed to explore whether adults in Canada with BMI in the overweight category are more likely to report higher well-being for other PMH measures and whether this varies after stratifying by factors such as sex to better understand the relationship between BMI in the overweight category and higher PWB score observed in this study.

Beyond these positive associations, we found that individuals reporting a mood disorder or anxiety disorder tended to have lower PWB than those not reporting these mental illnesses. Symptoms such as sadness, hopelessness, fatigue, lack of interest, negative self-perceptions, excessive worry, sleep disturbance, restlessness and difficulty concentrating may make it more difficult for individuals with internalizing disorders to experience PWB.^33^ Indeed, mood and anxiety disorders are associated with limitations in functioning and well-being.^33^,^34^ Moreover, lower scores on all six PWB components have been observed among individuals with depression (vs. without depression),^35^ and changes in depressive symptoms have been shown to predict changes in PWB (and vice versa) over a 16-year period among older adults.^36^ Despite the robust associations between reporting a mood or an anxiety disorder and having PWB in the current study, it is important to note that much of the variance in PWB remains unexplained. These results are in line with the dual-continuum model of mental health that conceptualizes PMH (e.g. PWB) and mental illness as related but distinct concepts, with each contributing to overall mental health.^37^,^38^

Individuals who indicated that most of their days were extremely or quite a bit stressful tended to report lower PWB than those who perceived less stress in their life. The cognitive appraisal of frequent and intense stressful transactions with the environment could hinder aspects of PWB, such as the sense that a person has autonomy or mastery over their environment, although we did not examine how well individuals thought they could cope with the level of stress in their lives.^39^ Nevertheless, this finding extends Canadian knowledge on the link between perceived stress and other PMH outcomes, including sense of community belonging, in the general adult population,^40^ and self-rated mental health and life satisfaction among adults with a mood and/or anxiety disorder.^41^ Ongoing promotion of PMH among individuals reporting high levels of stress or with mood or anxiety disorders may strengthen population PWB.^42^

In terms of substance use, engaging in heavy episodic drinking and in frequent cannabis use were significantly associated with lower PWB. Previous Canadian research with youth also found that heavy episodic drinking and frequent cannabis use were more common among those with low psychological and social well-being (albeit autonomy had a positive association with substance use when sociodemographic characteristics and other aspects of well-being were controlled for).^13^ Interestingly, smoking status was not associated with PWB in our study, whereas previous research found that recent cigarette use was associated with lower psychological and social well-being among youth in Canada.^12^ Inconsistent evidence for the relationship between smoking and aspects of PWB (i.e. purpose in life) have been noted previously.^43^ Future studies are needed to obtain a better understanding of the relationship between substance use and PMH by assessing motivations for use, examining polysubstance use and the use of other substances not measured in the current survey, taking into account group dynamics, distinguishing between those who abstain versus those who use at a low-risk level, examining associations with other PMH outcomes, and investigating the frequency of heavy episodic drinking.

Although we were able to identify multiple correlates of PWB, we also observed that some variables were not significantly associated with PWB. For instance, PWB differences across sex, education and immigrant status were not observed in this study, nor in analyses of the 2015 CCHS.^18^ PWB also did not differ between racialized versus non-racialized group members or for those with versus without children living at home. Differences in other PMH outcomes for these sociodemographic characteristics have been documented among adults in Canada,^24^ which highlights the importance of monitoring numerous PMH indicators. The lack of significant results for household income and major chronic disease status was unexpected. Previous analyses of the 2015 CCHS found that adults in the three highest household income quintiles had higher odds of high PWB than adults in the lowest household income quintile.^18^ The use of a more extreme reference group, the larger sample size and other methodological or analytical differences in the previous analyses may explain the conflicting results for household income. Previous Canadian research found that adults with chronic physical conditions were less likely to have flourishing mental health (albeit not after adjustment for various sociodemographic characteristics, positive health behaviours and chronic pain).^44^ More research is needed to understand these inconsistent findings.

***Strengths and limitations***

A key strength of this study includes the use of data from a population health survey that allowed us to examine a range of potential PWB correlates among community-dwelling adults living in the 10 Canadian provinces. In terms of limitations, PWB is multifaceted and has a range of potential correlates that were not measured or controlled for (e.g. personality),^7^ which could result in residual confounding. The use of self-reported data is subject to social desirability or recall biases, particularly for more sensitive content such as substance use.^45^ Although BMI was adjusted using sex-specific corrections to account for common reporting biases, some misclassification could have occurred due to the limitations of BMI for some populations (e.g. very muscular individuals, older adults, members of some racialized groups).^21^ The categorization of some of the explanatory variables in this study was broad (e.g. marital status, racialized group member); differences in PWB could have been missed between specific populations that were grouped together. Moreover, the findings of this study may not be generalizable to individuals who were excluded from the target population (e.g. those living in institutions or in Indigenous settlements, full-time members of the Canadian Armed Forces) or excluded from analyses (e.g. those aged less than 18 years, those who reported being pregnant). Lastly, given that the data analyzed were cross-sectional, causality and directionality of the observed associations cannot be ascertained.

## Conclusion

According to Ryff,^9^ PWB is experienced when people feel competent, self-determined and connected with others, when they have a sense of growth, purpose and meaning in life and when they accept who they are. We found that reporting a mood disorder, an anxiety disorder or high perceived life stress, and engaging in heavy episodic drinking and frequent cannabis use were consistently associated with lower PWB, whereas older age, being married or in a common-law relationship and having a BMI in the overweight category were consistently associated with higher PWB. Our results identify potential risk and protective factors of PWB, and highlight the need for future research on interventions aimed at increasing PWB. This is especially pertinent now given the decreased prevalence of other PMH outcomes (e.g. fewer adults reporting very good or excellent mental health),^46^ the increased prevalence of some potential risk factors (e.g. more adults screening positive for major depressive disorder; more adults reporting an increase versus a decrease in their alcohol and cannabis consumption)^47^,^48^ and larger inequalities in PMH outcomes among some groups (e.g. younger vs. older adults)^25^ that have been observed in Canada during the COVID-19 pandemic. It will be important to explore the degree to which PWB has also been impacted.

## Acknowledgements

The authors would like to thank Statistics Canada for their contribution to the design of the survey, data collection and data dissemination. We would like to thank the staff at the Data Coordination and Access Program (DCAP) at PHAC for their assistance with data dissemination. The authors would like to thank Karen C. Roberts (PHAC) and Natalie Gabora (PHAC) for reviewing the manuscript. Lastly, we would like to thank all of the people who participated in the 2019 CCHS Rapid Response on PMH.

## Conflicts of interest

Heather Orpana is one of this journal’s Associate Scientific Editors, but recused herself from the review process for this article.

The authors have no conflicts of interest to disclose.

## Authors’ contributions and statement

MV: Conceptualization, formal analysis, methodology, project administration, visualization, writing – original draft, writing– review & editing.

ZMC: Formal analysis, methodology, validation, writing – review & editing.

MMB: Conceptualization, writing – review & editing.

EP: Conceptualization, writing – review & editing.

HO: Conceptualization, writing – review & editing.

CAC: Formal analysis, methodology, validation, writing – review & editing.

The content and views expressed in this article are those of the authors and do not necessarily reflect those of the Government of Canada.
